# Treatment outcomes of dogs with transitional cell carcinoma

**DOI:** 10.3389/fvets.2025.1486786

**Published:** 2025-04-25

**Authors:** Ka To Chu, Omid Nekouei, Antonio Giuliano

**Affiliations:** ^1^Veterinary Medical Centre, City University of Hong Kong, Kowloon, Hong Kong SAR, China; ^2^Department of Infectious Diseases and Public Health, Jockey Club College of Veterinary Medicine and Life Sciences, City University of Hong Kong, Kowloon, Hong Kong SAR, China; ^3^Department of Veterinary Clinical Science, Jockey Club College of Veterinary Medicine and Life Sciences, City University of Hong Kong, Kowloon, Hong Kong SAR, China

**Keywords:** canine, chlorambucil, metronomic chemotherapy, TCC, transitional cell carcinoma, urinary bladder mass

## Abstract

Transitional cell carcinoma (TCC) is the most prevalent cancer of the urinary tract in dogs. The prognosis is often poor, and the optimal standard treatment has not been established. The objectives of this study were to (1) describe the clinical outcomes of dogs with TCC, and (2) determine the potential effects of tumor locations and treatment modalities on the survival times of patients. Electronic records of client-owned dogs with TCC treated with different modalities in a large veterinary hospital in Hong Kong (2005–2024) were evaluated. Of 84 confirmed cases included in the study, 49 (58.3%) died or were euthanized due to TCC. Tumors were located in the bladder neck or trigone region (41), apex (26), prostate (10), and urethra (7). Metastases were detected in 10 patients (12%) at diagnosis, including 4 peripheral lymph nodes, 4 lungs, and 2 in the lumbar spine. Of 84 cases, 4 (4.8%) did not receive any treatments, 14 (16.7%) underwent surgery, 25 (29.7%) received metronomic chemotherapy with chlorambucil with/without methotrexate, 27 (32.1%) received COX-2 inhibitors alone, and 14 (16.7%) received conventional chemotherapy, of which, 5 were later switched to metronomic chemotherapy. The overall median survival time was 233 days. There was no statistically significant difference in patients’ survival between tumor locations (*p* > 0.05), aside from tumors involving the prostate that had the shortest MST (88 days). Metronomic chemotherapy led to a significantly longer survival time (median of 303 days) than the other treatment groups (*p* < 0.05), with the lowest incidence of adverse events. Metronomic chemotherapy using chlorambucil was well-tolerated and can be considered as a single modality treatment or as adjunctive therapy to conventional chemotherapy in dogs with TCC.

## Introduction

Canine transitional cell carcinoma (TCC), also referred to as urothelial carcinoma, is the most common malignant neoplasm of the urinary tract in dogs, accounting for about 1.5 to 2% of all canine cancers (1, 2). Certain breeds, such as Scottish terriers, Shetland sheepdogs, and West Highland white terriers (1, 3) are predisposed to TCC (1, 3). The tumor is aggressive, invading the urinary bladder wall, and often metastasizes to the regional lymph nodes and distant organs, with the metastatic disease found in more than half of the patients at necroscopy (4, 5). However, most dogs are usually euthanized due to the progression of the local disease and/or lower urinary tract obstruction (6). The clinical signs of TCC are nonspecific and include haematuria, dysuria, pollakiuria, stranguria, and urinary obstruction (2, 4).

The prognosis of canine TCC is generally poor, with most patients surviving less than a year despite treatment (7, 8). The most common treatment options for canine TCC consist of chemotherapy often with mitoxantrone, carboplatin, gemcitabine, doxorubicin and vinblastine (9–15). Non-steroidal anti-inflammatory drugs (NSAIDs) with cyclooxygenase-2 (COX-2) inhibiting properties, especially piroxicam and meloxicam are often used alone or in combination with chemotherapy (11, 15–19). The role of surgery in the treatment of TCC is still controversial (4, 20). Most canine TCCs are located in the trigonal region of the urinary bladder where surgery is usually not feasible (10). Even when the tumor is located away from the trigone and resected with complete margins, recurrence is often seen due to urinary bladder field cancerization (21, 22). Despite surgery alone is unlikely to be effective, a combination of surgery and chemotherapy has shown some promising results (2, 4, 6–8, 11, 16, 17). Unfortunately, the aforementioned therapies are all accompanied by a common incidence of adverse events, impacting the patient’s quality of life, which is one of the major reasons patients were elected to be euthanized in some of the cases.

In addition to conventional chemotherapy agents, metronomic chemotherapy has emerged as a promising alternative or adjunctive treatment for canine TCC. Metronomic chemotherapy refers to a continuous and repetitive administration of a chemotherapeutic agent at a lowered dose, contrasting to a conventional regime, where agents are usually administered at maximum tolerated dose as boluses with longer intervals. Chlorambucil is an alkylating agent that inhibits DNA synthesis and functions by cross-linking with cellular DNA (23–25). It has direct anti-tumor effects, inhibiting angiogenesis and immunomodulatory properties that suppress tumor growth (23). In a prospective study on metronomic chemotherapy on canine TCC patients, chlorambucil showed 70% clinical benefit, with a good safety profile, as only 23% of dogs developed mild adverse events (25).

Currently, there is little data on canine tumor cell carcinoma and its treatment options in Asia, and no literature comparing the efficacy and adverse events of metronomic chlorambucil with other approaches. This retrospective study analyzed dogs with TCC treated at a Hong Kong veterinary hospital (2005–2024) to (1) describe their clinical outcomes and assess the impact of tumor location (bladder apex, bladder neck/trigone, prostate, urethra) and (2) treatment modalities (surgery, COX-2 inhibitors, conventional and metronomic chemotherapy) on patient survival.

## Methods

### Data collection

The electronic database of a tertiary referral veterinary hospital was searched for all canine patients diagnosed with TCC between January 2005 and March 2024. After complete data extraction, all patients meeting the following criteria were included in our study: (1) dogs with confirmed TCC diagnosis by a board-certified pathologist using cytology, histopathology, or CADET BRAF mutation test by a reference laboratory, (2) complete records including the date of birth and death, record of TCC diagnosis, record of surgery in our hospital or dispensation of TCC therapies, and (3) at least one revisit following the initial consultation on the medical records, with the documentation of adverse events between visit(s). Collected data included age at the time of diagnosis, breed, sex, neuter status, the location of TCC, type of treatment, dose, and number of chemotherapy treatments administered, observed adverse events, clinical response, objective response, and patient outcomes.

### Treatments

For statistical analysis, administered treatments to the study cases were categorized into five groups: (1) surgery plus COX-2 inhibitors, (2) conventional chemotherapy alone, (3) metronomic chemotherapy alone, (4) metronomic following conventional chemotherapy (patients who responded well to chemotherapy and their stable disease status were maintained with metronomic chemotherapy), and (5) COX-2 inhibitors alone.

Surgery consisted of a partial cystectomy to remove tumors with as wide margins as possible. Long-term COX-2 inhibitors were prescribed following surgery. The most common agent used was oral piroxicam (0.3 mg/kg once daily). Chemotherapy agents in this study included various intravenous administration of mitoxantrone (5–6 mg/m^2^ every 21 days), carboplatin (200–300 mg/m^2^, with a lower dose in smaller patients), and vinblastine (2 mg/m^2^). Based on the clinician’s preference, each protocol had chemotherapy agents in different orders. Metronomic chemotherapy consisted of the oral administration of chlorambucil at home, commonly in combination with a reduced dose of a COX-2 inhibitor. The chlorambucil dosage was 4 mg/m^2^ every 24 h, or every other day, often in combination with the standard dose of meloxicam 0.1 mg/kg, with/without the combination of oral methotrexate 2.5 mg weekly or biweekly, adjusted based on the weight of the dog and clinician’s preference. Metronomic following conventional chemotherapy referred to the group who switched to metronomic chemotherapy after the initiation of conventional chemotherapy. In the group of COX-2 inhibitors alone, oral piroxicam as a single agent (0.3 mg/kg once daily or every other day) was the most used agent. When administered in the metronomic setting, most dogs received meloxicam (0.1 mg/kg once daily), while the most common adjuvant COX-2 inhibitor given after surgery or a single agent was piroxicam (0.3 mg/kg once daily). Table 1 has summarized the treatment options for comparison.

**Table 1:** Summary of treatment groups.

Treatment group	Treatment description	Specific doses
Surgery	Partial cystectomy + long term oral COX-2 inhibitors	Oral piroxicam, 0.3 mg/kg SID
Conventional chemotherapy	Intravenous administration of a single or combination of chemotherapeutic agents	Mitoxantrone (5–6 mg/m^2^ every 21 days), carboplatin (200–300 mg/m^2^, with a lower dose in smaller patients), and vinblastine (2 mg/m^2^); variable
Metronomic chemotherapy	Oral administration of chlorambucil at home	Oral chlorambucil at 4 mg/m^2^ SID/EOD, +/− oral meloxicam 0.1 mg/kg SID, +/− oral methotrexate 2.5 mg weekly or biweekly
Metronomic following conventional chemotherapy	Metronomic chemotherapy after the completion of a full course of chemotherapy	As above
COX-2 inhibitors only	Utilize COX-2 inhibitors as the only therapeutic agents	Oral piroxicam as a single agent (0.3 mg/kg SID/EOD) or meloxicam (0.1 mg/kg SID) most commonly

### Tumor locations

The location of TCC is determined by imaging, most commonly with abdominal ultrasonography, sometimes with computed tomography when full staging was performed. Locations were categorized into four groups, namely bladder apex, neck, prostate, and urethra. If a tumor demonstrated a diffuse pattern or neoplastic lesions were found in more than one location, the largest measurable lesion represented the location of this tumor.

### Response to therapy

Evaluation of the response was based on the Veterinary Cooperative Oncology group’s RECIST response in solid tumors (26). The assessment of the response to treatment and subsequent monitoring were conducted using clinical examination, diagnostic imaging, and ultrasonography. The response was assessed by measuring the mass/masses/lesions before and during the regular follow-up. Complete response (CR) was defined as the resolution of all clinical and imaging-based evidence of the disease; partial response (PR) was defined as a decrease of at least 30% in tumor diameter with no new lesions; stable disease (SD) was defined as neither sufficient shrinkage to qualify as PR nor sufficient increase to qualify as progressive disease (PD). Progressive disease was defined as an increase in tumor diameter greater than 20% or the development of new lesions. Objective response rate (ORR) was defined as CR + PR. Clinical benefit rate (CBR) was defined as CR + PR + SD. A clinical response rate (CRR) was also calculated based on the improvement in clinical signs. For all patients included in the study, the progression-free survival was calculated in days from the date of the first chemotherapy treatment to the date of disease progression. Survival time for each patient was calculated from the TCC diagnosis to the date of death, euthanasia, or loss at follow-up. All observed adverse events were graded based on the Veterinary Cooperative Oncology Group Common Terminology Criteria for Adverse Events (VCOG-CTCAE v2) (27).

### Statistical analyses

All statistical analyses were conducted using Stata v18 (StataCorp LLC, College Station, United States). Descriptive statistics (e.g., median and range) for the survival times of patients were calculated and tabulated by the available explanatory variables. In survival analyses, death due to TCC was defined as the outcome of interest. Kaplan–Meier survival curves were created to visualize the survival of patients by tumor locations and treatment groups. The survival time and progression-free survival (PFS) were compared across tumor locations and treatment groups using the Wilcoxon–Breslow test. The PFS was defined as the time from the diagnosis until PD. Survival time was defined as the time from the diagnosis until death. To control the potential confounding effect of tumor location on treatment outcomes, the survival times of patients in different treatment groups were also compared in dogs that had TCC only in their bladder apex using the Wilcoxon-Breslow test.

## Results

Initially, 134 cases with suspected transitional cell carcinoma (TCC) were identified, but only 84 cases met the selection criteria. The majority of the excluded cases never had a definitive diagnosis nor returned for rechecks after the initial consultation. Cases without complete documentation of clinical response or tests performed were not included in this study. The frequency distribution of all cases by signalments, tumor location, and treatment modality has been presented in Table 2. Of the 84 patients, 46 were males (55%) and 38 females (45%), with 90.5% being neutered and 93% being purebred (Table 2). Patients were between 7 and 17.5 years old at the time of diagnosis, with a median of 12 years. Of the 84 patients, 41 had TCC in their bladder neck or trigone region, 26 in the apex, 10 in the prostate, and the remaining 7 had urethral involvements. Some forms of staging were performed in all cases but using different modalities. Abdominal ultrasound was performed in all cases. Thoracic radiography and CT-scan were conducted on 19 and 7 cases, respectively. Metastases were detected in 10 patients (12%) at diagnoses, of which, 4 had metastasis in peripheral lymph nodes, 4 had pulmonary metastasis and 2 had metastatic lesions in their lumbar spine. Among these 10 metastatic patients, 6 dogs had TCC in their trigone, 2 in the apex, and 2 in the prostate. A table showing all the patients, including but not limited to outcomes, treatment groups, location groups and adverse events, is provided as a Supplementary material to this study.

**Table 2:** Frequency distribution of 84 dogs with TCC in the study by available explanatory variables.

Variable	Category	Number	%
Sex/Neuter status	Spayed female	35	41.7
	Intact female	3	3.6
	Castrated male	41	48.8
	Intact male	5	6.0
Breed	Poodle	11	13.1
	Schnauzer	7	8.3
	Yorkshire terrier	7	8.3
	Shetland sheepdog	6	7.1
	Pomeranian	6	7.1
	Mongrel	6	7.1
	Shih Tzu	5	6.0
	Others^a^	36	42.9
Age group (year)	7 to <10	16	19.0
	10 to 13	39	46.4
	>13 to 17.5	20	23.8
Tumor location	Urethra	7	8.4
	Bladder neck/trigone	41	48.8
	Prostate	10	11.9
	Bladder apex	26	30.9
Treatment	No treatment	4	4.8
	Surgery	14	16.7
	Conventional chemotherapy	9	10.7
	Metronomic chemotherapy	25	29.8
	Metronomic following conventional chemotherapy	5	5.9
	COX-2 inhibitors	27	32.1

a Includes all other pure breeds with low numbers (<4).

### Patient outcome

**Figure 1:**
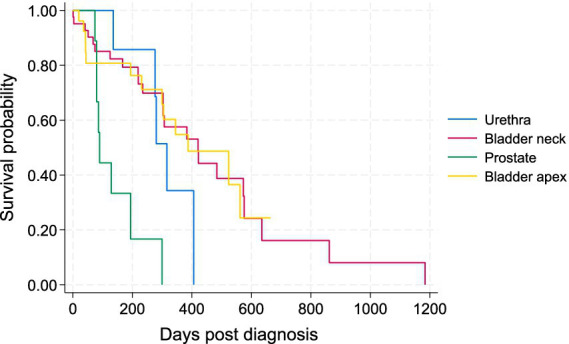
Kaplan–Meier survival curve for all 84 study dogs with TCC by tumor location.

Of the 84 dogs, 49 cases (58.3%) died or were euthanized due to TCC (11 with metastases and 38 due to the local progression). Twelve patients (14.3%) died of other diseases unrelated to TCC. Until the end of the study (March 2024), 10 patients (11.9%) were still alive, and 13 (15.5%) were lost to follow-up. The survival time of patients ranged between 1 and 1,184 days with a median of 233 days. The PFS ranged from 1 to 862 with a median of 177.5 days. The Kaplan–Meier survival curve for all patients by the four tumor locations is illustrated in Figure 1.

### Treatments

Of the 84 cases, 4 (4.8%) did not receive any treatments, and 25 (29.8%) received metronomic chemotherapy with chlorambucil, with/without methotrexate. Twenty-seven patients (32.1%) only received COX-2 inhibitors. Of the patients receiving metronomic chemotherapy, 82% also received COX-2 inhibitors. All 9 patients undergoing conventional chemotherapy (10.7%) also received adjunct COX-2 inhibitors. Five patients (5.9%) received metronomic chemotherapy following conventional chemotherapy. Among these 5 cases, 3 switched because of tumor progression, and 2 received metronomic when stable disease was achieved. Of the 14 patients undergoing surgery, 4 had incomplete excision and 10 had complete excision confirmed in histopathology. Of these 14 cases, 10 also received adjunct COX-2 inhibitors, and 4 received no other treatments.

Responses of all patients to the treatment groups are detailed in Table 3. Patients receiving conventional chemotherapy alone had an ORR of 22% and a CBR of 55%. For the metronomic chemotherapy group, ORR and CBR were 0 and 83.3%, respectively. For patients treated with COX-2 inhibitors alone, ORR was 7.4% and CBR was 85.2%. The five patients treated with metronomic chemotherapy following conventional chemotherapy had ORR and CBR of 0 and 40%, respectively. The CRR was 100% for all three chemotherapy groups, while CRRs for surgery and COX-2 inhibitor groups were 71.4 and 81.5%, respectively (Table 3). The owners of all 4 patients who received no treatments reported worsening clinical signs, one patient remained stable and 3 had progressive diseases.

**Table 3:** Responses of 84 study dogs with TCC to the four treatment groups.

Treatment^a^	No. of patients	Complete response (CR)	Partial response (PR)	Stable disease (SD)	Progressive disease (PD)	Objective response rate (ORR)	Clinical benefit rate (CBR)	Clinical response rate (CRR)
Conventional chemotherapy	9	0	2 (22.2%)	3 (33.3%)	4 (44.5%)	2 (22.2%)	5 (55.5%)	9 (100%)
Metronomic chemotherapy	25	0	0	22 (83.35)	3 (16.7%)	0	22 (83.3%)	25 (100%)
Metronomic following conventional chemotherapy	5	0	0	2 (40%)	3 (60%)	0	2 (40%)	5 (100%)
COX-2 inhibitors	27	0	2 (7.4%)	21 (77.8%)	4 (14.8%)	2 (7.4%)	23 (85.2%)	22 (81.5%)

a Surgery was not included in the table as the only outcomes were complete or incomplete excision, so response could not be measured.

### Adverse events

Among the 14 surgery patients, 8 (57.1%) developed gastrointestinal adverse events, including vomiting, diarrhea, melena and hyporexia (Veterinary Cooperative Oncology Group Common Terminology Criteria for Adverse Events Grade 1), 2 (14.3%) dogs died of postoperative complications, and the remaining 4 (28.6%) had no reported adverse events.

Of the 9 conventional chemotherapy cases, 2 (22.2%) developed Grade 1 GI adverse events, 3 (33.4%) had Grade 2 adverse events, such as hematemesis or mild neutropenia, and 2 (22.2%) developed Grade 3 adverse effects, such as lethargy or thrombocytopenia. The remaining 2 cases (22.2%) experienced no adverse events.

Of the 25 patients receiving metronomic chemotherapy, 12 (48%) dogs experienced no adverse events, 10 (40%) showed Grade 1 adverse events, 3 (12%) developed Grade 2 adverse events, including lethargy and listlessness, and no Grade 3 toxicity was reported. Of the 5 patients who received a combination of metronomic and conventional chemotherapy, 2 (40%) had Grade 1 adverse events, 1 (20%) had Grade 2 adverse events, 1 (20%) had Grade 3 adverse events, and 1 (20%) had no adverse events.

Among the 27 patients receiving COX-2 inhibitors alone, 12 (44.5%) dogs had no adverse events, 13 (48.1%) developed Grade 1 GI adverse events, 1 (3.7%) had Grade 2 adverse events, and 1 (3.7%) showed Grade 3 adverse events (azotemia).

### Location effects

**Figure 2:**
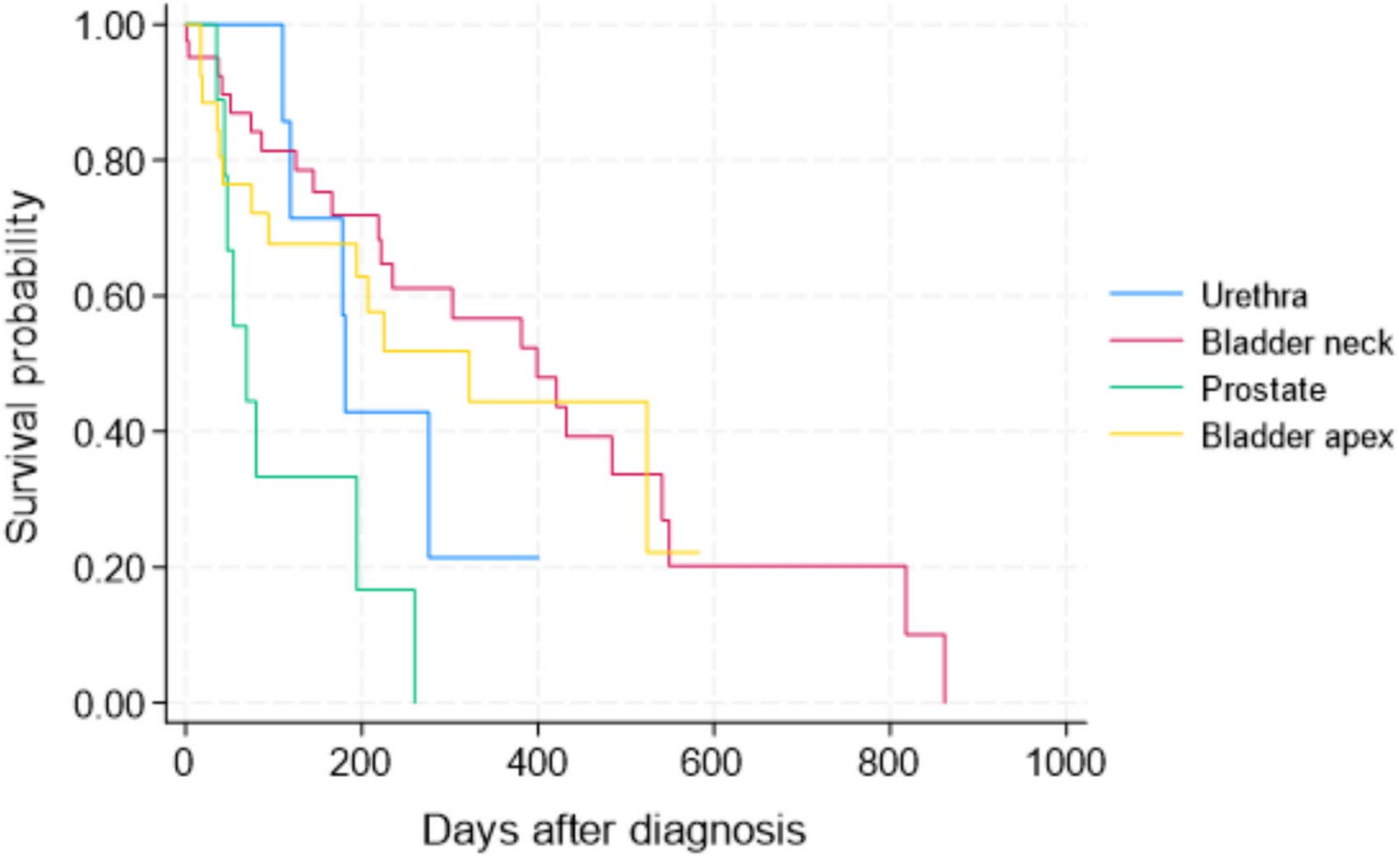
Kaplan–Meier progression-free survival (PFS) curve for all 84 study dogs with TCC by tumor location.

Patients with TCC in their prostates had significantly shorter survival times than those with tumors in the other three locations in the Wilcoxon tests (*p* < 0.05). There was no statistically significant difference in survival of patients between bladder neck/trigon, apex, and urethra (Figure 1). The Kaplan–Meier curve for PFS of patients by tumor location is depicted in Figure 2. The PFS of patients was significantly shorter in the prostate cases (PFS = 61.5 days) compared to the urethra and bladder neck (178 and 222 days, *p* = 0.044 and *p* = 0.003, respectively), but not significantly different than the apex (*p* = 0.104). There was no significant difference in PFS between the urethra, bladder neck, and apex (all *p* > 0.05).

**Figure 3:**
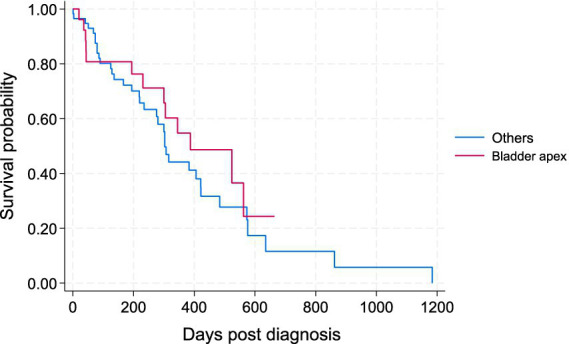
Kaplan–Meier survival curve for study dogs with TCC by two main locations; i.e., the apex of urinary bladder versus other locations (bladder neck, prostate, urethra).

The four tumor locations were further categorized into two main groups based on their likelihood for early urinary obstruction and to allow a more meaningful comparison: the apex of the bladder (the location considered less likely to cause an early urinary obstruction) versus other locations combined, including the bladder neck, prostate, and urethra (i.e., locations with a higher likelihood of causing early urinary obstruction) (Figure 3). There was no statistically significant difference in the survival of patients between the bladder apex and other locations in the Wilcoxon test (*p* = 0.573).

### Treatment effects

**Figure 4:**
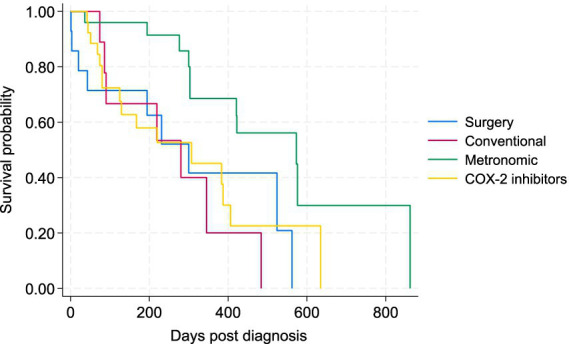
Kaplan–Meier survival curve for study dogs with TCC under four types of treatment, including surgery (plus COX-2 inhibitors), conventional chemotherapy alone, metronomic chemotherapy, and COX-2 inhibitors alone.

**Figure 5:**
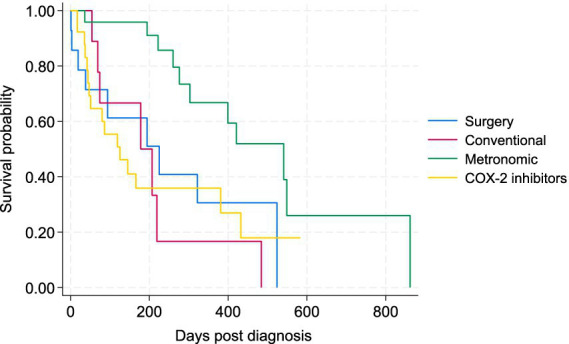
Kaplan–Meier progression-free survival (PFS) curve for study dogs with TCC under four types of treatment, including surgery (plus COX-2 inhibitors), conventional chemotherapy alone, metronomic chemotherapy, and COX-2 inhibitors alone.

To better assess the potential effects of treatments on the survival of patients, the 5 cases that received metronomic chemotherapy following conventional chemotherapy were removed from the comparisons, as there is potential carryover effect on the metronomic component from the conventional chemotherapy. The Kaplan–Meier survival curve for the four main treatment groups (surgery plus COX-2, conventional chemotherapy, metronomic chemotherapy, and COX-2 inhibitors alone) is presented in Figure 4. There was a statistically significant difference in the survival of patients between the metronomic chemotherapy and the other three treatment groups in the Wilcoxon tests (*p* < 0.05). There was no statistically significant difference between surgery, conventional chemotherapy alone, and COX-2 inhibitors groups (Figure 4). The Kaplan–Meier curve for PFS of patients under the four treatments is depicted in Figure 5. Similarly, the only statistically significant difference in PFS of patients was due to the difference between the metronomic chemotherapy group and the others (*p* < 0.05).

The medians of survival time and PFS for dogs who died of TCC are presented by tumor locations and treatments in Table 4, indicating MST was the longest for the three chemotherapy groups, especially for those dying after metronomic chemotherapy (303 days). Survival times for the 4 patients who did not receive any treatments were 41, 305, 276, and 316 days.

**Table 4:** Distribution of survival time (ST) and progression-free survival (PFS) of study dogs who died of TCC by tumor location and treatment group.

Variable	Category	No. of death	Median ST (range)	Median PFS (range)
Tumor location	Urethra	5	280 (136–406)	178 (110–276)
	Bladder neck/trigone	23	303 (1–1,184)	222 (1–862)
	Prostate	8	88 (74–300)	61.5 (35–260)
	Bladder apex	13	231 (20–562)	75 (17–524)
Treatment	Surgery (plus COX-2 inhibitors)	9	194 (1–562)	94 (1–524)
	Conventional chemotherapy	7	219 (74–484)	178 (54–484)
	Metronomic chemotherapy	11	303 (36–862)	303 (36–862)
	Metronomic following conventional	3	235 (136–1,184)	235 (110–818)
	COX-2 inhibitors	16	127 (42–635)	65.5 (17–432)

To account for any potential confounding effect of location, the survival time comparison between the four treatments was restricted to dogs with TCC on their bladder apex only (*n* = 25), no significant difference between the treatments was observed (*p* = 0.738).

## Discussion

This study compiled all canine TCC patients treated in a large veterinary hospital, including 84 confirmed cases treated with different modalities. We aimed to describe the clinical outcomes and assess the impact of tumor location and treatment modalities on patient survival.

In our study, metronomic chemotherapy using chlorambucil, with or without methotrexate, resulted in longer survival compared to other treatment options, with a median survival time (MST) of 303 days, consistent with findings reported in previous studies (25). As mentioned above, metronomic administration of chlorambucil offers anti-angiogenesis and immunomodulatory effects to reduce tumor growth. Growth of solid tumor is often accompanied by angiogenesis. A low-dose, continuous administration of chemotherapeutic agents was found to have sustained apoptotic effects on endothelial cells of tumors, thus disrupting and destroying the vascular tumor bed, achieving antitumor effects (28). Moreover, the immunomodulating effects of metronomic chemotherapy might potential benefit its antitumor function: by acting on immunosuppressive cells and allowing T cell and NK cells to infiltrate and attack tumors (29). Metronomic chlorambucil can offer TCC canine patients a promising alternative to traditional chemotherapy or COX-2 inhibitors. Furthermore, metronomic chemotherapy led to the longest PFS compared to other treatment options, indicating that it might offer longer survival in combination with a good quality of life. Potentially, dogs with advanced muscle invasive TCC can be considered a model for similar tumors in humans. Metronomic chemotherapy could also be investigated to be a treatment strategy that could be used in advanced bladder tumors in people when standard treatment have failed.

We initially expected that the combination of metronomic and conventional chemotherapy would result in longer survival than metronomic chemotherapy alone. However, our findings showed the opposite, with a shorter survival time in this group. Nonetheless, the small number of patients in the combined group could be the reason for this observation; therefore, we did not include this group in our direct statistical comparisons. The combined group only included 5 patients with 3 deaths, of which, 3 switched to metronomic chemotherapy due to progression or metastasis, suggesting a more aggressive tumor or a more advanced stage of disease that could have reduced the overall survival time in this group. The remaining 2 patients (of 5) that were switched to metronomic chemotherapy after achieving stable disease over conventional chemotherapy protocol had longer survival times of 401 and 1,184 days, and PFS of 401 and 818 days, respectively. Well-designed prospective studies with a larger number of cases are required to enable a robust comparison of patients’ outcomes between metronomic chemotherapy alone and a sequential combination of conventional and metronomic chemotherapy.

We hypothesized that the tumor location could affect the survival of patients with TCC. Iwasaki et al. (5) stated that patients with TCC in their bladder had significantly longer survival than urethra. In their study, urethral TCCs were found to have a significantly higher metastatic rate than TCCs in the bladder, thus shorter survival. Although full tumor staging was not performed in our patients, we can still hypothesize that TCC in the bladder apex may have a better prognosis than urethra and trigone as it is less likely to grow in an area of the bladder that would cause a more rapid urine outflow obstruction (10). Furthermore, complete surgical excision is more likely for apical masses compared to locations like the trigone. When comparing patient outcomes across different tumor locations, no significant difference in survival was observed. However, prostatic carcinomas had a significantly lower survival rate compared to tumors in other locations, a finding previously reported in the literature (1). It is often difficult to differentiate primary prostatic carcinoma from bladder TCC invading the prostate and it is possible that some of these tumors were prostatic adenocarcinoma rather than TCC. It is well known that prostatic adenocarcinoma has a poor prognosis both due to local rapid invasion causing early clinical signs of urinary obstruction and a higher metastatic rate (30). In our study, we also found that patients with prostate tumors had a higher metastatic rate (20%) compared to apex (7.7%), trigone (14.6%), and urethra (0%).

Tumor location could dictate the treatment offered by the clinicians (surgical versus non-surgical). To further account for any potential confounding effect of tumor location on the survival of treatment groups, we specifically compared the survival of patients between treatment groups only in apically located tumors which resulted in no significant difference in MST or PFS of the patients. The latter could suggest different types of treatment did not affect the survival of dogs with apical TCC, but this finding was not statistically robust and must be interpreted with caution due to the low number of cases within each treatment group (e.g., only one case in the conventional chemotherapy group) once we limited our data to the 26 cases of apical TCC only. When we specifically compare apically located masses with all other locations (non-apical masses), no significant difference in survival between the two main locations was found.

Because none of our surgery cases received adjuvant conventional or metronomic chemotherapy, we could not assess the potential additive effects of chemotherapy on surgery. According to Bradbury et al. (4), patients receiving a combination of partial cystectomy and medical therapy, which included chemotherapeutic agents and COX inhibitors, survived significantly longer than those who received medical therapy alone. Also, Molnár et al. reported that a combination of chemotherapy and surgery resulted in longer survival than chemotherapy or surgery alone (31). It would be interesting to compare our current treatment groups with surgical patients who also received chemotherapy, be it conventional or metronomic, and see if there is a survival benefit for a hybrid approach. Nonetheless, there are some risks of performing surgery in patients with TCC. Common complications of partial cystectomy in TCC post-operation include hematuria, pollakiuria, urinary incontinence, and dehiscence, with a reported complication rate of 43 to 81% in two studies (4, 32). Tumor seeding from partial cystectomy can also contribute to rapid regrowth or metastasis in patients who received surgery, leading to a poorer survival and outcome. One of the patients with trigonal TCC in this study was euthanized 1 day after surgery due to uroabdomen and dehiscence despite two revisit surgeries. There was another dog who received a partial cystectomy and passed away 3 days post-surgery at home due to an unknown reason. Another patient who had a complete excision, as confirmed by histopathology, died 20 days post-surgery due to immediate regrowth of the mass and spinal metastasis.

Most of the dogs who underwent surgical removal of TCC were administered COX-2 inhibitors as an adjuvant therapy. Theoretically, COX-2 inhibitors could delay the regrowth of the tumor by its antitumor effect as post-surgical adjuvant treatment (11). Although the MST of surgery plus COX-2 group (194 days) was higher than COX-2 alone (127 days) in our study, this difference was not statistically significant. Marvel et al. (20) reported that the MST of patients receiving daily administration of piroxicam combined with partial cystectomy reached 772 days, which was significantly longer than the survival times in TCC patients who underwent surgery alone. The paper pointed out that adding an adjuvant therapy to surgery might bring survival benefits to patients, but it is also worthwhile to consider the huge discrepancy in MST between our surgical patients and those in Marvel et al. Besides the difference in sample sizes, one of the theories is case selection. As previously mentioned, only a fraction of our sample was fully staged. There was a possibility that clients selected surgery for patients with metastasis or advanced disease, thus poor outcomes. In fact, one of the surgical patients in our study lived up to 562 days, which implied the importance of case selection when it comes to canine TCC (31). Considering the promising antitumoral effect of metronomic chemotherapy, future studies on the use of adjunct metronomic chemotherapy in surgical patients may offer an alternative to adjuvant COX-2 inhibitors alone.

Ravicini et al. (33) reported that dogs receiving a combination of chemotherapy and COX-2 inhibitors had significantly longer survival times than those receiving COX-2 inhibitors alone. Knapp et al. (11) stated that dogs receiving cisplatin had an MST of 338 days while dogs receiving only firocoxib had an MST of 152 days, but the difference was not statistically significant. Although not directly comparable, in a clinical trial by Schrempp et al. (25), the MST for dogs with TCC receiving chlorambucil was 221 days, whereas the MST of dogs receiving only COX-2 inhibitors in another study was 181 days (16). TCC often causes obstruction in locations such as trigone, ureter, or with invasion into the prostate, and most patients had deteriorated quality of life and were euthanized when obstruction occurred (9). Chemotherapy and metronomic chemotherapy offer better outcomes compared to COX-2 inhibitors alone. Nevertheless, COX-2 inhibitors remain a sensible choice of treatment for cases who cannot receive or decline chemotherapy.

Metronomic chemotherapy with chlorambucil with or without methotrexate led to the lowest occurrence of adverse events among all treatment groups in our study. In Schrempp et al. (25), metronomic chemotherapy with chlorambucil as a treatment for TCC yielded a low rate of adverse events of 23%. Despite a higher rate of adverse events in this study (52%), the majority of the adverse events are low-grade and no grade 3 or above adverse event was reported (25). In another prospective study by Leach et al. (23), using chlorambucil in treating different cancers, metronomic chemotherapy rarely caused adverse events, and they were limited to grade 1 or 2. Our study echoes the previous studies in terms of safety profile in long-term use in our canine patients. COX-2 inhibitors can cause adverse renal or GI events; for example, melena, hematochezia, and hematemesis, especially when piroxicam is used for a long time (34, 35). Conventional chemotherapy resulted in the highest adverse events, including Grade 3 adverse events, such as lethargy and neutropenia, which were often resolved by postponing the next dose of injection (36). The lower rate of adverse events following metronomic chemotherapy not only can lead to a better life quality in patients but also allows better continuity and client compliance without treatment delay, resulting in potentially better outcomes. In addition to a safer toxicity profile, metronomic chemotherapy entails easier oral administration than conventional injectable chemotherapies, thus reducing the number of treatments and revisits and consequently lowering costs. Nearly half of the patients who received metronomic chlorambucil also received oral methotrexate in our study. Methotrexate is an oral antimetabolite chemotherapy mainly excreted by the urine and used in people with bladder cancer (37). Despite methotrexate being given at low doses, concentration in the urine for a long period could increase the cancer control rate for bladder TCC in dogs. Nonetheless, the fact that only 12 patients were treated with this combination precluded robust statistical comparison; therefore, further studies are needed to verify this assumption.

In our study, metronomic chemotherapy was associated with longer survival. However, no statistically significant difference was found between other treatment groups, such as COX-2 inhibitors alone and conventional chemotherapy. The potential benefit of continuing low doses of chemotherapy instead of high pulsatile doses of conventional chemotherapy, in slow-growing tumors, such as the case in some bladder TCC, is reasonable. However, a larger prospective study comparing metronomic, conventional, and metronomic plus conventional chemotherapies is needed to validate our findings. It should be noted that chlorambucil had a poor ORR for canine TCC in our study. Indeed, none of the dogs receiving chlorambucil had a measurable response upon therapy, indicating that metronomic chemotherapy can effectively slow the tumor growth, but its efficacy in reducing the size in extensive disease with partial or complete obstruction is limited and the selection of the treatment on a case-by-case basis is paramount. In advanced TCC affecting the urethra/trigonal area, conventional chemotherapy could be more likely to produce a favorable outcome or measurable response (8, 11, 17).

We had a group of patients (*n* = 4) who received no treatment in this study. It is emphasized that treatment options were recommended for these cases based on each patient’s condition, but the owners declined. Under normal circumstances, no treatment is discouraged for animal welfare reasons. These 4 dogs had various survival times of 41, 305, 276, and 316 days. The relatively long survival times in three of these patients could have been due to being in the early stages of the disease. According to medical records, the patient who survived 41 days had evidence of metastasis in the lumbar spine whereas the remaining patients had no signs of metastasis on staging by radiography and ultrasonography.

There were some potential limitations to our study. The retrospective nature of the study does not allow for drawing strong inferences about the location and treatment effects and controlling for potential biases. As mentioned earlier, the low number of cases in some groups did not allow for robust statistical comparisons. For example, although we tried, we were not able to use more sophisticated survival analysis tests, such as the Cox proportional-hazards model to conduct multivariable analyses, as the underlying assumptions (e.g., proportional hazards) were not met. Staging and restaging of tumors were mainly done by ultrasound and the size and amount of urine were not standardized for comparison, hence the RR could have been under or overestimated. However, it should be noted that repeated CT scans for restaging, including general anesthesia, are rarely performed and carry extra costs for the owners. Although we have tried to eliminate the confounding effect of location and treatment, there is still a confounding factor we could not address due to the number of treatment categories and locations. Besides, the low number of observations with non-censored outcomes in some categories in age, breeds, and sub-categories made it impossible to completely eliminate confounders. Despite the outlined potential limitations, we believe including 84 TCC cases from nearly 20 years and handling the data with the ultimate care in this study have resulted in reliable and interesting findings, further contributing to the limited body of evidence on canine TCC topic and setting the stage for well-designed prospective studies (preferably clinical trials) addressing the highlighted gaps.

In conclusion, prostatic TCCs had significantly shorter survival time than TCC in other locations. Metronomic chemotherapy led to longer survival and PFS and the lowest occurrences of adverse events compared to other treatment options in our study. For tumors located in the bladder apical region, surgery in combination with COX-2 inhibitors resulted in comparable survival to metronomic chemotherapy. Metronomic chemotherapy had relatively lower costs as a long-term control for canine TCC. While metronomic chemotherapy is promising as a relatively safe and effective treatment option for canine TCC, well-designed clinical trials are still recommended to establish the most effective treatment option/s by specific tumor location.

## Data Availability

The raw data supporting the conclusions of this article will be made available by the authors, without undue reservation.
