# Supplementary material for: Treatment outcomes of dogs with transitional cell carcinoma
Source: Front Vet Sci. 2025 Apr 25;12:1486786. doi: 10.3389/fvets.2025.1486786 (PMC12063355; doi:10.3389/fvets.2025.1486786)
Supplement: Supplementary file 1 [file Table_1.DOCX]

| **Patient** | **Age at diagnosis (years)** | **Sex** | **Neuter status** | **Breed** | **Weight at diagnosis (kg)** | **Location** | **Treatment** | **Response** | **Adverse events** | **Progression Free Survival** | **Survival Time** | **Outcome** |
| --- | --- | --- | --- | --- | --- | --- | --- | --- | --- | --- | --- | --- |
| 1 | 15 | M | N | Yorkshire terrier | 3 | 3 | 0 | NA | 1 | 322 | 562 | 0 |
| 2 | 16 | F | S | Shih Tzu | 4 | 1 | 4 | SD | 1 | 86 | 220 | 0 |
| 3 | 12 | F | S | Shih Tzu | 5 | 3 | 0 | NA | 1 | 413 | 413 | 3 |
| 4 | 12 | F | S | Shih Tzu | 7 | 1 | 4 | SD | 0 | 135 | 228 | 2 |
| 5 | 9 | M | N | Mini Collie | 13 | 1 | 4 | SD | 0 | 125 | 125 | 0 |
| 6 | 14 | M | N | Shih Tzu | 6 | 3 | 4 | PD | 1 | 17 | 44 | 0 |
| 7 | 11 | F | S | Mongrel | 21 | 3 | 0 | NA | 1 | 38 | 43 | 0 |
| 8 | 9 | M | N | Yorkshire terrier | 5 | 3 | 0 | NA | 1 | 19 | 20 | 0 |
| 9 | 15 | M | N | Cocker spaniel | 13 | 1 | 2 | SD | 0 | 148 | 317 | 3 |
| 10 | 14 | F | S | Yorkshire terrier | 3 | 3 | 4 | SD | 1 | 17 | 387 | 0 |
| 11 | 10 | F | S | Shih Tzu | 8 | 3 | 2 | PR | 3 | 207 | 345 | 0 |
| 12 | 14 | F | S | Mongrel | 22 | 1 | 2 | PD | 0 | 74 | 74 | 0 |
| 13 | 12 | M | N | Pekingese | 4 | 3 | 4 | SD | 0 | 42 | 42 | 0 |
| 14 | 12 | F | S | Cocker spaniel | 13 | 1 | 4 | SD | 1 | 166 | 167 | 0 |
| 15 | 12 | F | S | American cocker spaniel | 11 | 1 | 4 | SD | 0 | 145 | 307 | 0 |
| 16 | 14 | M | N | Bichon Frise | 5 | 2 | 4 | SD | 2 | 47 | 80 | 0 |
| 17 | 12 | F | E | Pomeranian | 4 | 1 | 4 | SD | 3 | 260 | 260 | 3 |
| 18 | 10 | M | E | Schnauzer | 10 | 1 | 0 | NA | 0 | 59 | 59 | 3 |
| 19 | 12 | F | S | Maltese X | 4 | 1 | 4 | SD | 1 | 33 | 51 | 3 |
| 20 | 14 | F | S | Mini collie | 9 | 1 | 2 | PR | 1 | 484 | 484 | 0 |
| 21 | 9 | M | E | Golden retriever | 26 | 2 | 2 | PD | 3 | 69 | 86 | 0 |
| 22 | 11 | F | S | Pomeranian | 4 | 3 | 0 | NA | 1 | 225 | 231 | 0 |
| 23 | 11 | F | S | Pekingese | 5 | 0 | 4 | SD | 1 | 119 | 406 | 0 |
| 24 | 10 | F | S | Samoyed | 26 | 3 | 0 | NA | 0 | 194 | 194 | 0 |
| 25 | 13 | M | N | Beagle | 15 | 2 | 4 | SD | 1 | 35 | 129 | 0 |
| 26 | 12 | M | N | Scottish terrier | 8 | 3 | 0 | NA | 0 | 524 | 524 | 0 |
| 27 | 13 | M | N | Schnauzer | 13 | 3 | 0 | NA | 1 | 94 | 300 | 0 |
| 28 | 11 | M | N | Shetland sheepdog | 10 | 1 | 4 | SD | 1 | 432 | 635 | 0 |
| 29 | 8 | F | S | Schnauzer | 11 | 1 | 2 | SD | 2 | 219 | 219 | 0 |
| 30 | 14 | F | E | Schnauzer | 8 | 1 | 5 | SD | 1 | 818 | 1184 | 0 |
| 31 | 10 | F | S | West highland terrier | 38 | 3 | 0 | NA | 0 | 481 | 490 | 3 |
| 32 | 8 | M | N | Pomeranian | 7 | 3 | 1 | NA | NA | 75 | 305 | 0 |
| 33 | 12 | M | E | Golden retriever | 26 | 1 | 4 | PD | 0 | 37 | 68 | 0 |
| 34 | 13 | M | N | Poodle | 7 | 3 | 0 | NA | 1 | 67 | 151 | 1 |
| 35 | 10 | M | N | Schnauzer | 8 | 2 | 4 | SD | 0 | 80 | 80 | 0 |
| 36 | 12 | F | S | Pug | 7 | 1 | 0 | NA | 5 | 3 | 3 | 0 |
| 37 | 15 | M | N | Mini Pinscher | 4 | 2 | 4 | SD | 1 | 44 | 74 | 0 |
| 38 | 14 | M | N | Chihuahua | 4 | 3 | 4 | PR | 1 | 583 | 583 | 3 |
| 39 | 11 | M | N | Mini collie | 9 | 1 | 4 | SD | 1 | 51 | 51 | 0 |
| 40 | 14 | M | N | Poodle | 9 | 3 | 3 | SD | 0 | 217 | 217 | 3 |
| 41 | 12 | M | N | Shetland sheepdog | 11 | 3 | 3 | SD | 1 | 373 | 514 | 1 |
| 42 | 11 | M | N | Mini Collie | 10 | 1 | 5 | PD | 3 | 235 | 235 | 0 |
| 43 | 11 | M | N | Shetland sheepdog | 11 | 1 | 1 | NA | NA | 216 | 276 | 1 |
| 44 | 8 | F | S | Poodle | 4 | 1 | 3 | SD | 2 | 399 | 421 | 0 |
| 45 | 10 | M | N | Bichon frise | 7 | 1 | 4 | SD | 0 | 34 | 94 | 1 |
| 46 | 13 | M | N | poodle | 8 | 1 | 3 | SD | 0 | 436 | 436 | 2 |
| 47 | 11 | M | E | Shetland sheepdog | 19 | 2 | 3 | SD | 1 | 260 | 300 | 0 |
| 48 | 11 | M | N | Border collie | 17 | 3 | 3 | PD | 0 | 105 | 105 | 3 |
| 49 | 12 | M | N | Shetland sheepdog | 11 | 0 | 1 | NA | NA | 181 | 316 | 0 |
| 50 | 14 | M | N | Pug | 10 | 1 | 3 | SD | 1 | 549 | 573 | 2 |
| 51 | 8 | M | N | Yorkshire terrier | 3 | 0 | 4 | SD | 1 | 212 | 212 | 3 |
| 52 | 14 | F | S | Collie | 16 | 1 | 0 | NA | 5 | 1 | 1 | 0 |
| 53 | 12 | F | S | Maltese | 4 | 3 | 3 | SD | 0 | 511 | 664 | 1 |
| 54 | 13 | F | S | Pomeranian X | 7 | 1 | 3 | SD | 1 | 222 | 303 | 0 |
| 55 | 15 | F | S | Chihuahua | 3 | 1 | 1 | NA | NA | 41 | 41 | 0 |
| 56 | 12 | F | S | Maltese | 4 | 3 | 3 | SD | 0 | 196 | 357 | 1 |
| 57 | 12 | M | N | Yorkshire terrier | 6 | 3 | 4 | PD | 0 | 32 | 92 | 1 |
| 58 | 15 | M | N | Poodle | 5 | 3 | 0 | NA | 1 | 90 | 210 | 1 |
| 59 | 13 | M | N | Corgi | 12 | 1 | 4 | PD | 1 | 24 | 84 | 1 |
| 60 | 10 | F | E | Pomeranian | 6 | 1 | 4 | SD | 0 | 381 | 383 | 0 |
| 61 | 11 | M | N | Poodle | 3 | 1 | 4 | PR | 0 | 506 | 506 | 2 |
| 62 | 12 | M | N | Mongrel | 24 | 1 | 3 | SD | 2 | 862 | 862 | 0 |
| 63 | 8 | F | S | Poodle | 4 | 1 | 3 | SD | 1 | 421 | 422 | 0 |
| 64 | 7 | F | S | Mongrel | 29 | 1 | 3 | SD | 1 | 541 | 576 | 0 |
| 65 | 11 | F | S | Mongrel | 16 | 0 | 5 | SD | 1 | 401 | 401 | 2 |
| 66 | 10 | F | S | Yorkshire terrier | 2 | 3 | 4 | SD | 0 | 273 | 273 | 2 |
| 67 | 7 | F | S | Sheltie | 16 | 0 | 5 | PD | 0 | 110 | 136 | 0 |
| 68 | 9 | M | N | Corgi | 19 | 2 | 4 | SD | 0 | 2 | 2 | 2 |
| 69 | 10 | M | N | Dachshund | 6 | 2 | 2 | PD | 1 | 54 | 90 | 0 |
| 70 | 13 | M | N | Poodle | 8 | 1 | 3 | SD | 0 | 437 | 437 | 2 |
| 71 | 12 | F | S | Schnauzer | 7 | 1 | 2 | PD | 2 | 131 | 131 | 2 |
| 72 | 12 | M | N | Scottish Terrier | 8 | 0 | 2 | SD | 2 | 178 | 280 | 0 |
| 73 | 11 | M | E | Shetland Sheepdog | 21 | 3 | 3 | PD | 1 | 36 | 36 | 0 |
| 74 | 14 | F | S | Poodle | 6 | 1 | 3 | SD | 0 | 236 | 236 | 3 |
| 75 | 9 | M | N | Poodle | 39 | 2 | 5 | PD | 2 | 177 | 177 | 2 |
| 76 | 11 | M | N | Mongrel | 42 | 2 | 3 | SD | 1 | 194 | 194 | 0 |
| 77 | 9 | F | S | Welsh Corgi | 17 | 1 | 3 | SD | 0 | 137 | 137 | 2 |
| 78 | 14 | M | N | Pug | 10 | 1 | 3 | SD | 0 | 549 | 573 | 0 |
| 79 | 15 | F | S | Schnauzer | 6 | 0 | 3 | SD | 0 | 276 | 276 | 0 |
| 80 | 18 | F | S | Dachshund | 7 | 1 | 3 | SD | 0 | 126 | 126 | 3 |
| 81 | 13 | F | S | Pomeranian X | 7 | 1 | 3 | SD | 2 | 303 | 303 | 0 |
| 82 | 13 | M | N | Poodle | 12 | 3 | 3 | SD | 1 | 286 | 468 | 1 |
| 83 | 11 | F | S | Bichon Frise | 10 | 1 | 3 | PD | 0 | 35 | 226 | 2 |
| 84 | 11 | M | N | Yorkshire Terrier | 3 | 1 | 3 | SD | 1 | 242 | 242 | 2 |

Note:

For location, 0 is urethra; 1 is bladder neck/trigone; 2 is prostate; 3 is bladder apex.

For treatment, 0 is surgery; 1 is no treatment; 2 is conventional chemotherapy; 3 is metronomic chemotherapy; 4 is COX-2 inhibitor; 5 is metronomic chemotherapy following conventional chemotherapy

For AE, numbers refer to VCOG-CTCAE v2 AE grading; 0 means no AE.

For outcome, 0 is deaths due to TCC; 1 refers to patients still being alive; 2 is lost to follow-up; 3 is deaths of other causes.
